# Structural Coloration of Polyester Fabrics Coated with Al/TiO_2_ Composite Films and Their Anti-Ultraviolet Properties

**DOI:** 10.3390/ma11061011

**Published:** 2018-06-14

**Authors:** Xiaohong Yuan, Yuanjing Ye, Min Lian, Qufu Wei

**Affiliations:** 1 Fujian Key Laboratory of Novel Functional Textile Fibers and Materials, Faculty of Clothing and Design, Minjiang University, Fuzhou 350108, China; xm6844264@126.com; 2 Fujian Fibers Inspection Bureau, Fuzhou 350026, China; dick1999@126.com; 3 Key Laboratory of Eco-Textiles, Ministry of Education, Jiangnan University, Wuxi 214122, China; qufuwei@jiangnan.edu.cn

**Keywords:** structural coloration, Al/TiO_2_ composite films, anti-ultraviolet property, magnetron sputtering

## Abstract

Al/TiO_2_ composite film was successfully deposited on polyester fabrics by using magnetron sputtering techniques. X-ray photoelectron spectroscopy (XPS) and X-ray diffraction (XRD) were used to examine the deposited films on the fabrics, and the structural colors and anti-ultraviolet property of fabrics were also analyzed. The results indicated that polyester fabrics coated with Al/TiO_2_ composite films achieved structural colors. The reactive sputtering times of TiO_2_ films in Al/TiO_2_ composite films were 10 min, 12 min, 18 min, 20 min, 26 min, 27 min, 30 min and 45 min, respectively, the colors of corresponding fabrics were bluish violet, blue, cyan, green, yellow, yellowish red, orange and blue-green, which was consistent with the principle of the thin film interference. The structure of the TiO_2_ film in Al/TiO_2_ composite films was non-crystalline, though the fabrics were heated and maintained at the temperature of 200 °C. The anti-ultraviolet property of the fabrics deposited with Al/TiO_2_ composite films were excellent because of the effect of Al/TiO_2_ composite films.

## 1. Introduction

With the intensification of pollution problems in the traditional printing and dyeing industry and the strengthening of people’s awareness of environmental protection, structural coloration, as an ecological dyeing technique, has attracted increasing attention from researchers [1,2,3,4,5]. Structural coloration, differing from chemical dyeing, without water and chemicals, utilizes the dispersion, scattering, diffraction and interference of light to create colors [6]. Therefore, research interest in structurally-colored textiles has gradually increased and is currently mainly focused on photonic crystals materials. However, the preparation process of photonic crystal materials is rather complicated and the cost is high, so it is difficult to realize industrial production [7,8,9,10]. Our research group developed a simple and low cost method to prepare structurally-colored textile, that is, textiles coated with nano-metal/semiconductor composite films by magnetron sputtering technology, not only can achieve the structure colors on the textiles surface, but also obtain other functions [11]. In our previous study, the metal silver was chosen as the metal film material. In this paper, the metal aluminum (Al) was chosen as the metal film material.

Metal aluminum is very common in nature, and its price is much cheaper than metal Ag. Aluminum has many excellent properties, such as conductivity, anti-ultraviolet, anti-electromagnetic shielding and other properties. The Al film is the only material in the metal film that has a high reflectance from the ultraviolet, visible to infrared, and has excellent optical properties [12,13]. However, there are few reports on the Al/TiO_2_ composite films deposited [14,15], especially on the fabric substrates.

Metal Al materials and semiconductor TiO_2_ materials were selected in this paper, and nano-Al/TiO_2_ composite films were deposited on the fabric surface by magnetron sputtering technology. The structural colors and anti-ultraviolet property of fabrics coated with nano-Al/TiO_2_ composite films were analyzed. This research is intended to provide a theoretical and experimental basis for the development of multi-functional and structurally-colored textiles.

## 2. Experimental Section

### 2.1. Materials

The substrate used was a white polyester plain weave fabric. The fabric was cut into a circular sample with a diameter of 5 cm, and then washed with the acetone solution, followed by drying and storing.

The sputtering targets used were 99.99% Aluminum (Al) target and 99.99% titanium (Ti) target.

### 2.2. Preparation of Al/TiO_2_ Composite Films

Al/TiO_2_ composite films were prepared by the sputtering unit (JPG-450 type, SKY Technology Development Co., Ltd., Shenyang, China). The working conditions were set as a base pressure of 1.5 × 10^−3^ Pa and a working gas pressure of 0.8 Pa. 99.99% Argon was used a working gas and the distance between the target and the fabric substrate was 70 cm. The revolving speed of samples was 10 r/min in order to achieve the even deposition on the fabric. The preparation process of Al/TiO_2_ composite films was as follows.

Firstly, Al film was deposited by RF magnetron sputtering with an Argon (Ar) gas flow rate of 20 mL/min, a sputtering power of 120 W and for a sputtering time of 30 min. Then the titanium film was deposited by DC magnetron sputtering with an Ar gas flow rate of 50 mL/min, a sputtering power of 100 W and for a sputtering time of 10 min. Finally, the titanium dioxide film was deposited by RF reactive sputtering using titanium target with a sputtering power of 300 W, gas flow rates of Ar and O_2_ were set as 20 mL/min and 10 mL/min respectively. The sputtering time was set as 10 min, 12 min, 18 min, 20 min, 26 min, 27 min, 30 min and 45 min, respectively, and the corresponding sample numbers was marked as Nos. 1, 2, 3, 4, 5, 6, 7 and 8.

### 2.3. Microstructure and Composition of Al/TiO_2_ Composite Films

The chemical composition and valence state of the deposited composite films on the textile substrate were analyzed by X-ray photoelectron spectroscopy (Escalab 250XiXPS, VG Instruments, UK) using Al Ka monochromator as X-ray source.

The composite films deposited on the textile substrate also were examined by X-ray diffraction (D8XRD, Bruker-AXS, Karlsruhe, Germany) measurements on a Bruker-AXS X-ray diffractometer system with Cu Kα radiation. Scanning range was 2–90°.

### 2.4. Structural Color and Color Fastness Test

The color photographs of all samples were taken with a Japanese Sony DCR-HC90E digital camera.

The colors of the samples were analyzed using a spectrophotometer (Color-Eye 7000A, GretagMacbeth, USA). According to the 1976 norm of the Commission International de I’Eclairage (CIE), light source of D65, observation angle of 10°, L*, a*, b*, C* values and reflectance curves were tested respectively. Among them, the lightness L*, the chromaticity a* and b* refer to three mutually perpendicular coordinate axes in the color space, and C* is the chroma, which indicates the purity of the color. The reflectance curve also reflects the color of the sample.

The fastness of composite films deposited on polyester fabrics could be expressed by color fastness to washing, which was tested according to GB/T 3921-2008 [16] “textile test for color fastness to washing with soap or soap and soda”.

### 2.5. Anti-Ultraviolet Property Test

According to GB/T18830-2009 [17], anti-ultraviolet properties of the samples were tested by ultraviolet transmittance analyzer (UV-1000F, Lapsphere, USA).The evaluation indexes of anti-ultraviolet properties included solar ultraviolet transmittance T (UVA) and T (UVB) and ultraviolet protection factor (UPF), solar UV-A spectral transmittance T (UVA), solar UV-B spectral transmittance T (UVB) and ultraviolet protection factor (UPF). Each sample was tested five times, and the average values were reported.

## 3. Results and Discussion

### 3.1. XPS Analysis

**Figure 1:**
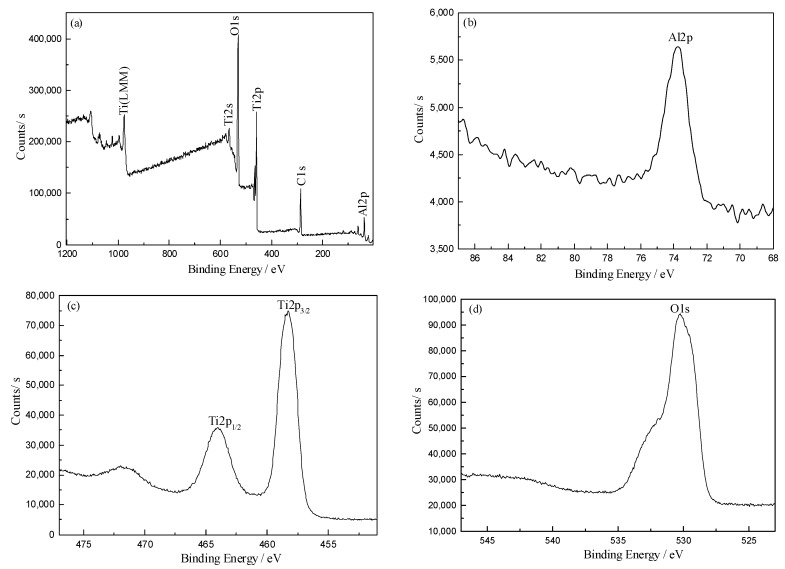
X-ray photoelectron spectroscopy of No. 4 sample: (**a**) Full spectrum; (**b**) Al2p peak; (**c**) Ti2p peak; (**d**) O1s peak.

All samples coated with Al/TiO_2_ composite films differ only in the thickness of TiO_2_ film. Therefore, the XPS test results should be similar, and No. 4 sample was selected as a representative to analyze its chemical composition and valence state. Figure 1 presents the photoelectron spectroscopy of No. 4 sample.

In Figure 1a, only the characteristic peaks of Al, Ti, and O elements are presented in the XPS full spectrum of No. 4 sample, and there was also a handful of C, which is mainly derived from the carbon in the ultra-high vacuum chamber in the XPS equipment. Figure 1b shows the Al2p peak of Al, and it can be seen that the position of Al2p peak was 73.74 eV, which is approximately the same as the binding energy of metal Al, suggesting that Al was present in the composite films in the form of metallic aluminum [18,19]. As can be seen from Figure 1c, the positions of Ti2p_1/2_ and Ti2p_3/2_ peak were 464.00 eV and 458.28 eV, respectively, and the results were consistent with the TiO_2_ binding energy. It indicates that Ti was completely oxidized, and was presented in TiO_2_ form [20,21]. Figure 1d shows the O1s peak of the O element. It can be seen from Figure 1d that the O1s peak position of O element was located at 530.07 eV, and the result is consistent with the binding energy of O in TiO_2_ [22,23]. It also indicates that Ti existed in the composite film in the form of TiO_2_.

Consequently, the XPS analysis indicates that the composite films deposited on the polyester fabric surface were Al/TiO_2_ composite films.

### 3.2. XRD Analysis

Due to Al/TiO_2_ composite films were prepared by magnetron sputtering at room temperature, the TiO_2_ film in the composite films was difficult to form a crystal structure. In general, TiO_2_ film was crystallized by sputtering at high temperature or calcination after sputtering [24].

**Figure 2:**
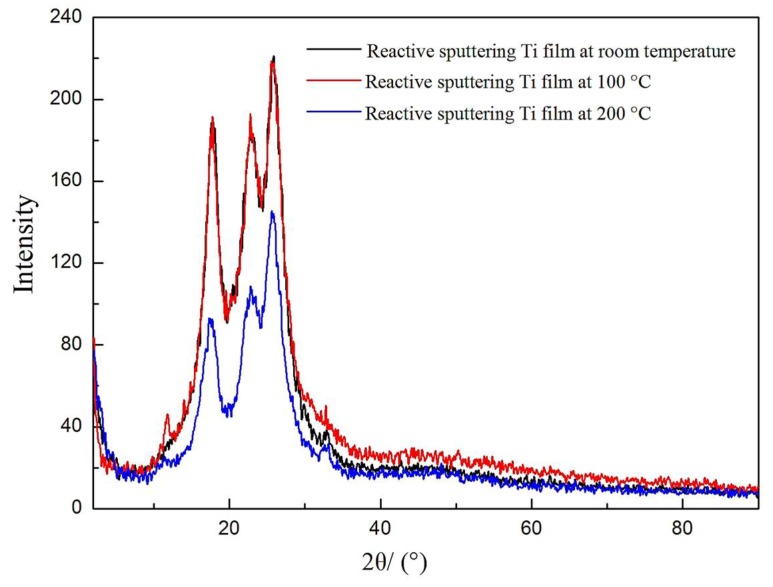
XRD patterns of the sample coated with Al/TiO_2_ composite films for reaction sputtering TiO_2_ film at different temperature.

Without affecting the properties of the textile substrate, the temperature of the textile fabric substrate was raised during reactive sputtering so as to crystallize the TiO_2_ films in the Al/TiO_2_ composite films. Since the experimental substrate was polyester fabrics, too high a temperature would affect the fabric dimensional stability and performance. Therefore, the fabric substrate was heated and maintained the temperature at 100 °C and 200 °C respectively during RF reactive sputtering. Taking No. 7 as an example, the XRD test was performed when the temperature of the textile substrate was normal temperature, 100 °C, and 200 °C, respectively. The experimental results are shown in Figure 2.

It can be seen from Figure 2 that the XRD patterns of the Al/TiO_2_ composite films deposited on polyester fabrics prepared by reactive sputtered TiO_2_ film at room temperature, 100 °C and 200 °C look similar. There were only the characteristic peaks of polyester fabric substrate, without the characteristic peaks of the crystalline structures of Al and TiO_2_, indicating that the structures of Al and TiO_2_ all were amorphous structures.

Normally, the metal element was easy to form a crystal structure, but the Al film in the Al/TiO_2_ composite film was not crystallized here. The main reason was that the preparation of the Al film used RF sputtering, and the deposition rate was very low, which affected the ordered arrangement of atoms, resulting in the amorphous structures structure of Al. The type of anatase crystal of TiO_2_ film was generally obtained for at temperatures between 350 °C and 500 °C, and the type of rutile crystal was formed when the temperature exceeded 500 °C [25,26]. Considering the influence of polyester fabric substrate, the temperature was only added to 200 °C, resulting in a non-crystalline structure of TiO_2_ film.

To summarize, the XRD patterns of Al/TiO_2_ composite films deposited on polyester fabrics prepared by reactive sputtering of TiO_2_ film at room temperature, 100 °C and 200 °C indicated that both Al and TiO_2_ in the Al/TiO_2_ composite films had non-crystalline structures. TiO_2_ film cannot form a crystal structure at the temperature of 200 °C, but 200 °C was the limit temperature for heating the polyester fabrics, while the performance of polyester fabrics were affected by further heating.

### 3.3. Structural Color Analysis

**Figure 3:**
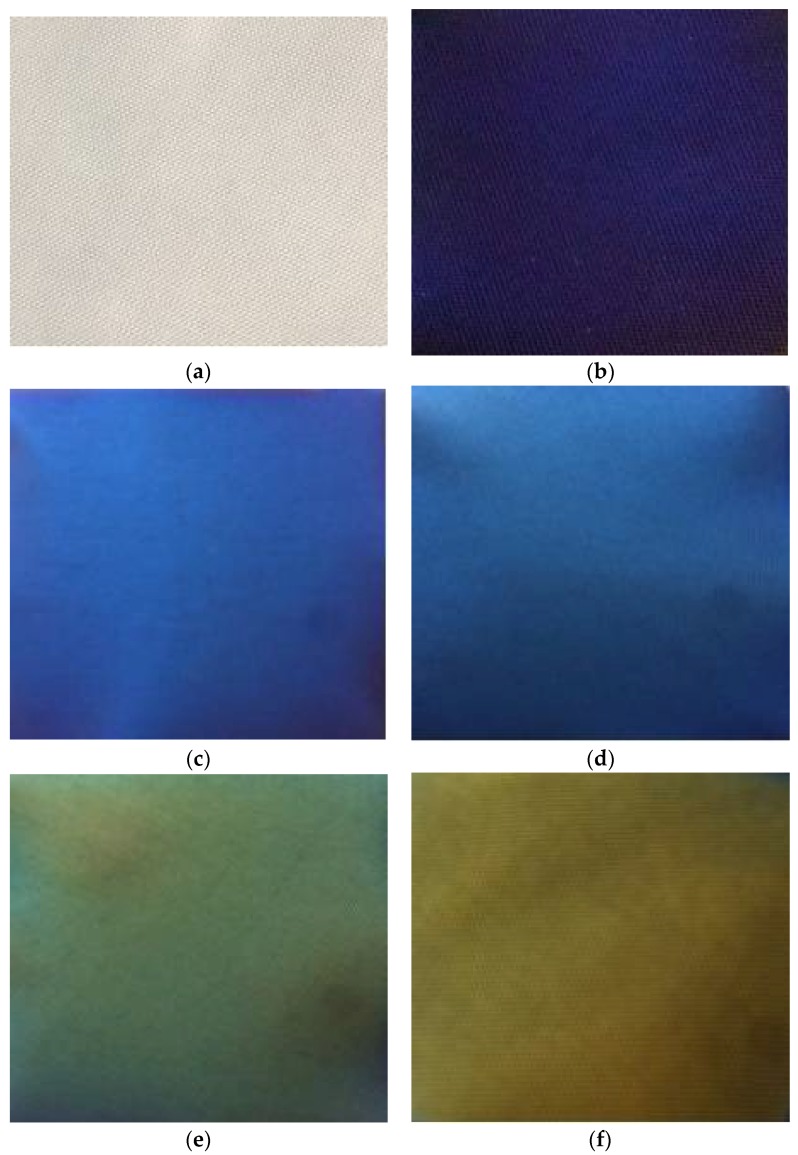

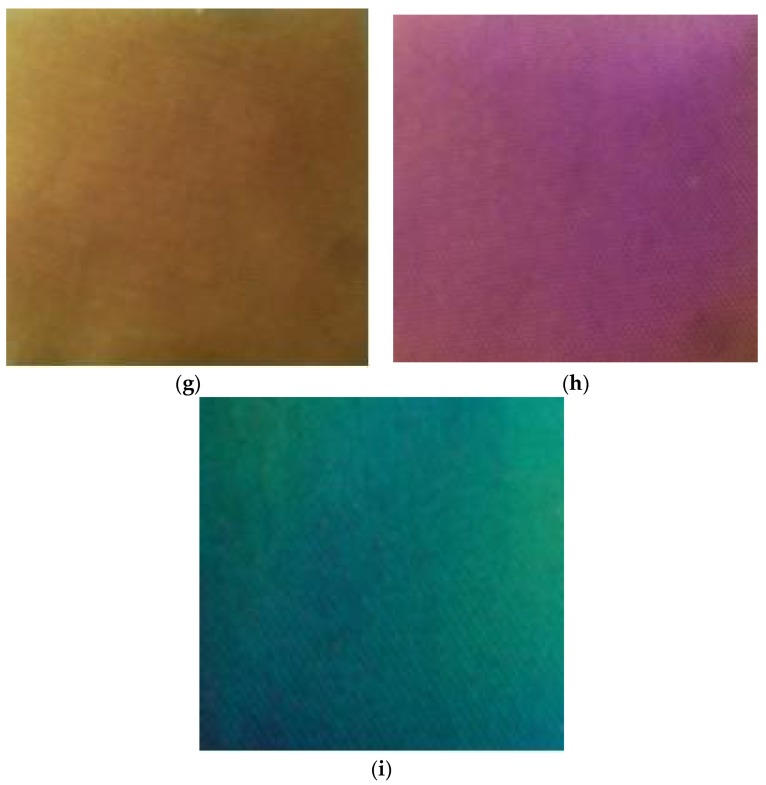
Color photographs of the original sample and the samples with different sputtering time of TiO_2_ film: (**a**) Original sample; (**b**) 10 min; (**c**) 12 min; (**d**) 18 min; (**e**) 20 min; (**f**) 26 min; (**g**) 27 min; (**h**) 30 min; (**i**) 45 min.

The color photos of the original fabric and the polyester fabrics coated with Al/TiO_2_ composite film are shown in Figure 3. These photos were formed by selecting 2 cm from the central of samples and then enlarging.

It can be clearly seen from Figure 3 that the thickness of the TiO_2_ film in Al/TiO_2_ composite films was different and the colors of the samples were also different. The original white fabric presented a variety of structural colors because their surfaces were coated with Al/TiO_2_ composite films. As the thickness of TiO_2_ films in Al/TiO_2_ composite films increased, the structural colors changed from purple, blue, cyan, green, yellow, orange to red, respectively. In this experiment, the thickness of the TiO_2_ film was mainly controlled by the reactive sputtering time. The reactive sputtering times of TiO_2_ films for Nos. 1–8 samples were 10 min, 12 min, 18 min, 20 min, 26 min, 27 min, 30 min and 45 min, respectively, and the corresponding fabric colors were bluish violet, blue, cyan, green, yellow, yellowish red, orange and blue-green. The results indicated that the corresponding wavelength of the samples was proportional to the thickness of the TiO_2_ film, which was consistent with the thin film interference theory.

**Figure 4:**
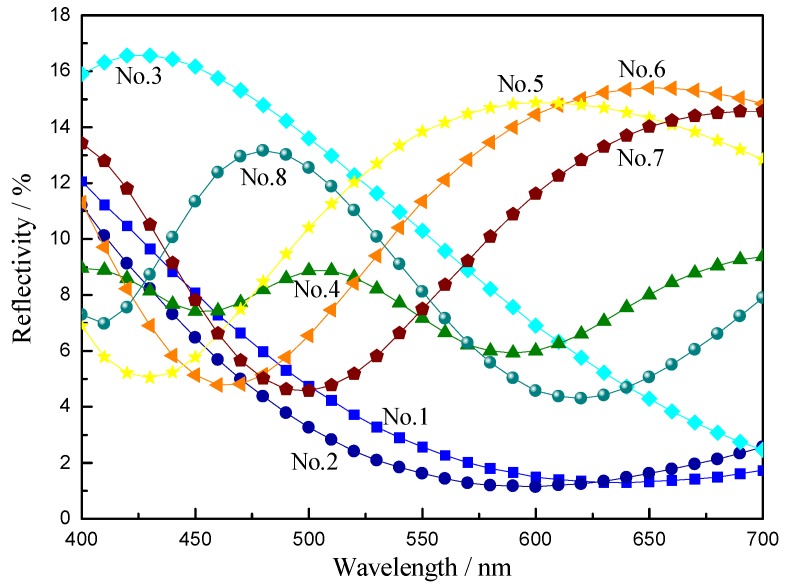
Reflection spectra of the samples coated with Al/TiO_2_ composite films.

Figure 4 reveals the corresponding reflection spectra of samples coated with different thickness of TiO_2_ thin films in Al/TiO_2_ composite film.

The spectral reflectance curves of Nos. 1 and 2 samples were close to each other, but they were slightly offset. The maximum reflection wavelength was approximate 400 nm, which is in the purple and blue wavelength range. Therefore, the colors of Nos. 1 and 2 samples were determined to be bluish violet and blue, consistent with the fabric colors presented in Figure 3b,c. The center wavelength of the reflectivity curve of the No. 3 sample was located at 420 nm, which is in the blue wavelength range. Therefore, the No. 3 sample color was cyan, which is also consistent with the fabric color in Figure 3d. The maximum reflection wavelength of No. 4 sample was 510 nm, which is in the green wavelength range, and was therefore consistent with the fabric color in Figure 3e. The maximum reflection wavelengths of No. 5 sample, No. 6 sample and No. 7 sample were 600 nm, 650 nm, and 690 nm, respectively corresponding to yellow, yellowish red, orange color, and consistent with the results in Figure 3f,g,h. The maximum reflection wavelength of No. 8 sample was 480 nm, corresponding to blue-green color, and consistent with the result in Figure 3i.

L*, a*, b* and C* values for samples are shown in Table 1.

From Table 1, it can be seen that the L* values of Nos. 1–8 samples were quite different, indicating that the lightness of the samples was significantly distinct. The chroma C* values of Nos. 1–8 samples differed greatly, with the minimum chroma value of No.4 and the maximum chroma value of No. 2 sample, also manifesting that the color and purity of the samples are different. The a* and b* values of Nos. 1–8 samples were different, indicating that the samples colors were in different positions in the color space.

**Figure 5:**
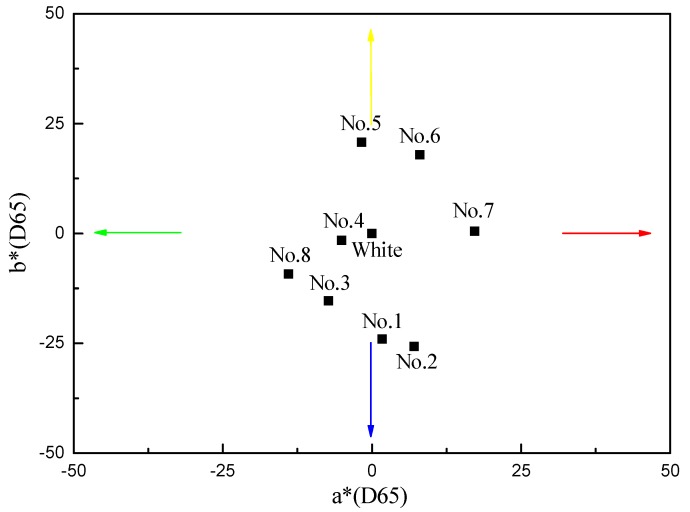
Distribution of chromaticity indices, a* and b*, for the samples coated with Al/TiO_2_ composite films.

Figure 5 represents the distribution of chromaticity indices, a* and b*, for all the samples coated with Al/TiO_2_ composite films.

As shown in Figure 5, a* and b* values of Nos. 1–8 samples coated with Al/TiO_2_ composite films were different in the color space. According to the CIE 1976 chromaticity diagram, the colors of these samples were bluish violet, blue, cyan, green, yellow, yellowish red, orange and blue-green. The results are consistent with the experimental results shown in Figure 3 and Figure 4.

As a consequence, polyester fabrics coated with Al/TiO_2_ composite films presented structural colors resulted from the effect of nano-composite films, and the colors varied along with the change of the TiO_2_ film thickness. With the increase of the TiO_2_ film thickness, the colors of fabrics changed from purple, blue, cyan, green, yellow, orange to red. When the thicknesses of the TiO_2_ films continued to increase, the colors of the fabrics changed regularly followed by these seven colors, and the results were consistent with the rules of thin film interference. In this experiment, the reactive sputtering times of TiO_2_ films for the samples were 10 min, 12 min, 18 min, 20 min, 26 min, 27 min, 30 min and 45 min, respectively, and the corresponding fabric colors were bluish violet, blue, cyan, green, yellow, yellowish red, orange and blue-green.

Compared with the polyester fabrics coated with Ag/TiO_2_ composite film samples, the color rules of fabrics coated with nano-composite films were the same, though the underlying metal in composite films was different. The corresponding wavelength of colors was linearly proportional to the thickness of TiO_2_ films [27].

Due to the different refractive indexes of metallic Ag and Al, the sputtering efficiency of the TiO_2_ film was affected, resulting in the difference between the sputtering time and the corresponding wavelength of the fabric color. For example, the reactive sputtering times of the TiO_2_ films in Ag/TiO_2_ composite films deposited on polyester fabrics were 1 min, 3 min, and 4 min, correspondingly, the reactive sputtering times of the TiO_2_ films in Al/TiO_2_ composite films deposited on polyester fabrics were 12 min, 20 min, and 26 min, respectively, the corresponding fabrics colors were blue, green, and yellow [25]. As a result, the deposition efficiency of the TiO_2_ films in Al/TiO_2_ composite films was lower than that in Ag/TiO_2_ composite films.

According to the experimental results, the color fastness to washing of polyester fabrics coated with Al/TiO_2_ composite films was 5, so the color fastness to washing of samples was very good. It indicated that Al/TiO_2_ composite films and polyester fabrics were combined strongly, and the composite films were firm, and not easy to fall off.

### 3.4. Anti-Ultraviolet Property Analysis

Table 2 shows the anti-ultraviolet properties of Nos. 1–8 samples coated with Al/TiO_2_ composite films. The smaller the values of T (UVA) and T (UVB), the anti-ultraviolet property is better. Conversely, the greater the ultraviolet protection factor (UPF), the anti-ultraviolet property is higher.

As can be seen from Table 2, the results of anti-ultraviolet property for Nos. 1–8 samples were similar, and T (UVA) and T (UVB) were less than 4%, and UPF was more than 30.

According to the national standard GB/T18830-2002 [28], when UPF > 30 and T (UVA) < 5%, the samples can be called anti-UV textiles. It can be seen that all the polyester fabrics coated with Al/TiO_2_ composite films meet the requirements and belong to the UV protection product. The main reason was that the high reflectivity of the Al film reflects ultraviolet light, and the TiO_2_ film in the Al/TiO_2_ composite film also has the effect of UV protection. Duo to high refractive and high photoactivity, nano-TiO_2_, as an excellent ultraviolet protection agent, can absorb ultraviolet ray, and reflect and scatter ultraviolet rays, and transmit visible light.

In summary, the anti-ultraviolet property of polyester fabrics coated with Al/TiO_2_ composite films can be greatly improved resulting from the influence of Al/TiO_2_ composite films.

## 4. Conclusions

Al/TiO_2_ composite film was successfully deposited on polyester fabrics using the magnetron sputtering technique. Due to the effect of nano-composite films, polyester fabrics coated with Al/TiO_2_ composite films achieves structure coloration. The reactive sputtering times of TiO_2_ films in Al/TiO_2_ composite films were 10 min, 12 min, 18 min, 20 min, 26 min, 27 min, 30 min and 45 min, respectively, the colors of corresponding fabrics were bluish violet, blue, cyan, green, yellow, yellowish red, orange and blue-green, which was consistent with the rules of the thin film interference. XPS analysis shows that the composited films deposited on the surface of the polyester fabrics substrate were Al/TiO_2_ composite films. The XRD patterns of Al/TiO_2_ composite films deposited on polyester fabrics prepared by reactive sputtering of TiO_2_ film at room temperature, 100 °C and 200 °C indicated that the structures of Al films and TiO_2_ films in Al/TiO_2_ composite films were non-crytalline structures. The anti-ultraviolet property of polyester fabrics coated with Al/TiO_2_ composite films were all excellent resulting from the influence of Al/TiO_2_ composite films.

## Figures and Tables

**Table 1:** L*, a*, b*, C* scale of the samples coated with Al/TiO_2_ composite films.

Samples	L*	a*	b*	C*
No. 1	19.781	1.681	−23.979	24.038
No. 2	15.710	7.106	−25.764	26.726
No. 3	37.515	−7.281	−15.323	16.965
No. 4	32.794	−5.054	−1.578	5.294
No. 5	43.093	−1.776	20.803	20.878
No. 6	39.357	8.058	17.917	19.646
No. 7	34.164	17.240	0.494	17.247
No. 8	34.174	−13.992	−9.235	16.764

**Table 2:** Anti-ultraviolet property of the samples coated with Al/TiO_2_ composite films.

Samples	T (UVA)/%		T (UVB)/%		UPF	
	Average Value/%	Standard Deviation/%	Average Value/%	Standard Deviation/%	Average Value	Standard Deviation
No. 1	3.65	0.44	3.30	0.14	31.92	0.88
No. 2	3.05	0.75	2.69	0.12	36.42	0.75
No. 3	3.75	0.12	3.45	0.09	30.66	0.68
No. 4	3.61	0.11	3.30	0.07	31.83	0.77
No. 5	3.49	0.08	3.22	0.10	32.74	0.98
No. 6	3.47	0.15	3.06	0.10	33.05	1.01
No. 7	3.82	0.05	3.41	0.05	30.82	0.86
No. 8	3.49	0.10	3.01	0.08	33.52	0.58
